# Prognostic factors in malaria patients with acute kidney injury: a systematic review and meta analysis

**DOI:** 10.1080/0886022X.2026.2665050

**Published:** 2026-05-25

**Authors:** Alveron Andreas Tear, Nisa Fauziah, Silvita Fitri Riswari, Amelia Reta Purba, Muhammad Makarimal Akhlaq, Nugroho Harry Susanto

**Affiliations:** a Department of Biomedical Sciences, Faculty of Medicine, Universitas Padjadjaran, Sumedang, Indonesia; b Division of Parasitology, Department of Basic Biomedical Science, Faculty of Medicine, Universitas Padjadjaran, Bandung, Indonesia; c Research Center for Care and Control of Infectious Diseases (RC3ID), Universitas Padjadjaran, Sumedang, Indonesia; d Clinical Research Unit, Prof. Dr. Mahar Mardjono National Brain Center Hospital, Jakarta, Indonesia

**Keywords:** Acute kidney injury, malaria, meta-analysis, mortality, prognostic factors

## Abstract

This systematic review and meta-analysis aims to evaluate the predictors of mortality in malaria patients with AKI. Studies were searched in PubMed, Scopus, Ebsco, and ScienceDirect. We included observational studies that showed the association between clinical or laboratory abnormalities and mortality, reported in odds ratio. The quality of each study was assessed using the Newcastle-Ottawa Scale. Meta-analysis was performed using R. Sixteen studies with 1,104 patients were analyzed. Factors significantly associated with mortality were respiratory distress syndrome (OR: 18.50, *p* < 0.001), neurological involvement (OR: 12.83, *p* < 0.001), disseminated intravascular coagulation (OR: 12.00, *p* < 0.001), hypotension (OR: 18.23, *p* < 0.001), oliguria (OR: 3.41, *p* = 0.002), ventilator requirement (OR: 30.35, *p* < 0.001), leukocytosis (OR: 4.87, *p* = 0.003), hyponatremia (OR: 3.67, *p* = 0.048), acidosis (OR: 4.88, *p* < 0.001), hyperkalemia (OR: 3.99, *p* < 0.001), and jaundice (OR: 5.03, *p* = 0.001). ARDS, neurological involvement, DIC, hypotension, oliguria, ventilator requirement, leukocytosis, hyponatremia, acidosis, hyperkalemia, and jaundice were associated with mortality in malaria patients with AKI. The findings underscored the importance of heightened awareness and vigilance in managing patients with these conditions.

## 1. Introduction

Malaria constitutes a significant public health challenge in tropical nations. The most recent World Malaria Report states that 263 million cases were recorded in 2023 compared to 252 million cases in 2022. In 2022, malaria caused approximately 608,000 deaths worldwide, with a mortality rate of 14.3 deaths per 100,000 people at risk [1]. As a result, the statistics associated with the incidence and mortality rates of malaria for the latest report are concerning.

Deaths from malaria often occur in patients with severe malaria [2]. This condition can present with various symptoms, including severe anemia, metabolic acidosis, cerebral malaria, jaundice, and acute kidney injury. One of the most common clinical manifestations in patients with severe malaria is AKI, which has a prevalence of 45.6% [3]. The mortality rate associated with AKI in malaria cases can reach up to 75%. A recent systematic review and meta-analysis found that AKI has the strongest association with mortality among clinical manifestations of severe malaria (odds ratio: 5.96, 95% confidence interval 2.93–12.11) [4,5]. Therefore, it is essential to understand the clinical manifestations and laboratory parameters related to the mortality of malaria patients experiencing these complications.

Previous studies indicate that jaundice, respiratory distress, oliguria, and disseminated intravascular coagulation negatively affect the prognosis of malaria patients with AKI. However, these findings remain contradictory and inconsistent across studies [6–21]. As such, further investigation is necessary, making a systematic review titled ‘Prognostic Factors in Malaria Patients with Acute Kidney Injury: a Systematic Review and Meta Analysis’ a worthwhile endeavor.

## 2. Material and methods

### 2.1. Data source and search strategy

**Figure 1.**
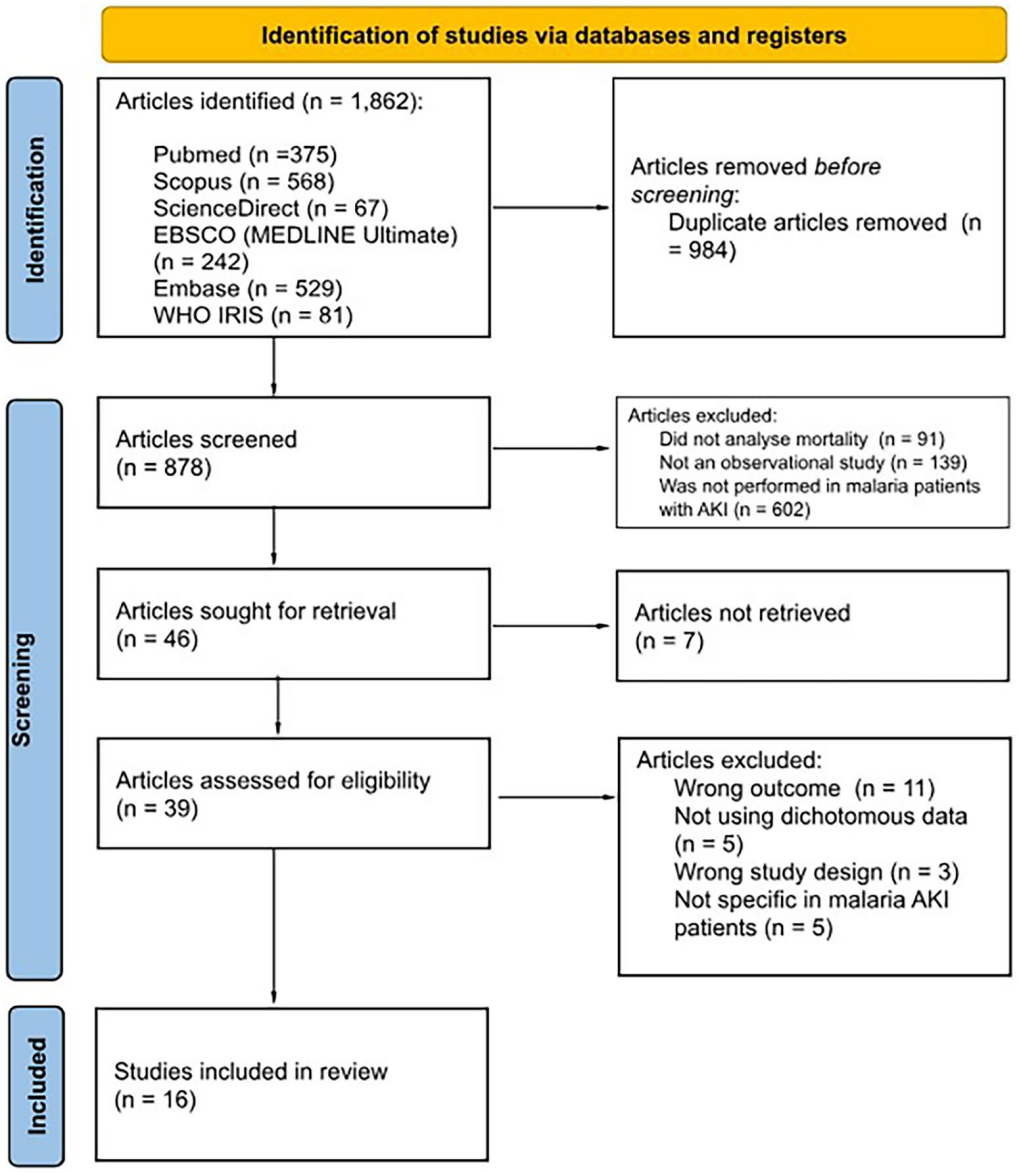
Flow diagram of included studies based on PRISMA flow char.

The protocol for this systematic review was registered in the international prospective register of systematic reviews (PROSPERO: CRD42024607978). The present review was executed according to the Preferred Reporting Items for Systematic Reviews and Meta-Analyses (PRISMA) (Figure 1). PubMed, Scopus, Ebsco, and ScienceDirect were searched for papers until 12 December 2025. The following concepts were used: ‘Malaria,’ ‘Acute Kidney Injury,’ and ‘Mortality.’ The complete searches can be accessed in the supplementary Table S1.

### 2.2. Study selection

Two reviewers (AAT and ARP) independently reviewed and selected the articles. Any discrepancy in articles selection will be resolved by the help of a third reviewer (NF). The inclusion criteria were: (1) Observational studies (cohort, case control) in malaria patients with AKI; (2) showed the association between abnormal clinical manifestation or laboratory values with mortality; (3) Odds ratio (OR) with 95% confidence interval (Cl) was directly reported or provide sufficient data to be calculated. Exclusion criteria: (1) Languages other than in English; (2) Studies that were not specific in malaria patients with AKI; (3) Studies that reported composite outcomes (not specific about mortality); (4) Inaccessible full text.

### 2.3. Data extraction and risk of bias

Two reviewers (AAT and ARP) independently extracted the data from included studies. The following were extracted: first author, study design, year of publication, study site, age, number of participants, Plasmodium species, and the potential prognostic factors. The quality of each study was assessed using the Newcastle-Ottawa Scale (NOS), which has a maximum of nine stars. The quality of each study was classified according to the Agency for Healthcare Research and Quality (AHRQ). Studies were considered good quality (3–4 stars in the selection domain, 1–2 stars in the comparability domain, and 2–3 stars in the outcome/exposure domain), fair quality (2 stars in the selection domain, 1–2 stars in the comparability domain, and 2–3 stars in the outcome/exposure domain), and poor quality (0–1 star in the selection domain, 0 stars in the comparability domain, or 0–1 stars in the outcome/exposure domain) [22].

### 2.4. Statistical analysis

Meta-analysis was conducted with R ver. 2024.12.1 + 563 (R Foundation for Statistical Computing, Vienna, Austria). Odds ratio (OR) with their respective 95% Cl were used to analyze the association. Multivariate/adjusted OR were preferentially extracted. When OR was not reported, univariate/crude OR was independently calculated using a two-by-two table. The meta-analysis was performed using a random-effects inverse-variance model and the DerSimonian and Laird method. The inconsistency index (I^2^ statistic) was used to assess study heterogeneity, with p-values < 0.05 considered statistically significant. A leave one out sensitivity analysis was performed to confirm the robustness of the result. If the number of studies that analyzed certain factors was greater than 10, publication bias analysis using funnel plot, egger’s, and begg’s tests will be performed.

## 3. Results

### 3.1. Study characteristics

The search resulted in 1,862 studies from the database. Initially, 984 duplicates were removed, resulting in 878 unique records screened for title and abstract. After the studies were screened, 46 records were assessed in full text. Eventually, only 16 articles were included in this review (Figure 1) [6–21].

The included studies were published between 1996 and 2018, with a total of 1,104 patients. Geographically, the studies originated from India, Pakistan, and Thailand. Seven studies were prospective cohorts [6,7,10,12,16–18] and nine studies were retrospective cohorts [8,9,11,13–15,19–21]. Eleven studies were performed in adults [10,12–14,17–19], three were performed in children [8,11,15], and six were performed in mixed populations [6,7,9,16,20,21]. Three were performed in *P. falciparum* infected patients [8,13,21], three were performed in *P. vivax* infected patients [9,12,18], and ten were performed in mixed patients [6,7,10,11,14–17,19,20]. The median NOS score was 7. Nine studies were judged to have good quality [8–10,12,14,16–19], seven were considered to have poor quality [6,7,11,13,15,20,21] (Table 1). To further explore the observed heterogeneity and the potential impact of study quality, subgroup analyses were performed based on predefined variables. The detailed results of these analyses are provided in the Supplementary Figures.

**Table 1.** Characteristic of included studies.

Author	Country	Study design	Sample size	Population	Study duration (Year)	Plasmodium species	Data type	NOS score	Quality
Anghan et al. 2018 [6]	India	Prospective Cohort	36	Mixed (patients >12 years)	2012–2014	Mixed *P. falciparum* and *P. vivax*	Univariate	7	Poor
Nigam et al. 2018 [7]	India	Prospective Cohort	101	Mixed (patients >14 years)	2016–2017	Mixed *P. falciparum* and *P. vivax*	Univariate	7	Poor
Prasad et al. 2016 [8]	India	Retrospective Cohort	31	Children (2–14 years)	2010–2013	*P. falciparum*	Univariate	7	Good
Naqvi et al. 2015 [9]	Pakistan	Retrospective Cohort	109	Mixed (8–78 years)	1990–2014	*P. vivax*	Univariate	7	Good
Shukla et al. 2013 [10]	India	Prospective Cohort	101	Adult (patients >18 years)	2010–2011	Mixed *P. falciparum* and *P. vivax*	Univariate	9	Good
Zaki et al. 2013 [11]	India	Retrospective Cohort	12	Children (1 month-12 years)	2009	Mixed *P. falciparum* and *P. vivax*	Univariate	6	Poor
Kaushik et al. 2013 [12]	India	Prospective Cohort	63	Adult (patients >18 years	2010–2011	*P. vivax*	Univariate	8	Good
Thanachartwet et al. 2013 [13]	Thailand	Retrospective Cohort	113	Adult (patients >15 years)	2004–2008	*P. falciparum*	Multivariate (Leukocytosis, Mechanical ventilation)	6	Poor
Khan et al. 2013 [14]	India	Retrospective Cohort	100	Adult (mean age: 30 years)	2010–2011	Mixed *P. falciparum* and *P. vivax*	Univariate	7	Good
Kapoor et al. 2012 [15]	India	Retrospective Cohort	18	Children (mean age: 75 months)	2010–2011	Mixed *P. falciparum* and *P. vivax*	Univariate	6	Poor
Kute et al. 2012 [16]	India	Prospective Cohort	59	Mixed (6–75 years)	2010	Mixed *P. falciparum* and *P. vivax*	Univariate	8	Good
Gupta et al. 2012 [17]	India	Prospective Cohort	74	Adult (15–65 years)	2003	Mixed *P. falciparum* and *P. vivax*	Univariate	8	Good
Kute et al. 2012 [18]	India	Prospective Cohort	25	Adult (mean age: 30 years)	2010–2011	*P. vivax*	Univariate	8	Good
Kanodia et al. 2010 [19]	India	Retrospective Cohort	100	Adult (mean age: 32 years)	2008–2010	Mixed *P. falciparum* and *P. vivax*	Univariate	7	Good
Naqvi et al. 2003 [20]	Pakistan	Retrospective Cohort	124	Mixed (5–75 years)	1990–1999	Mixed *P. falciparum* and *P. vivax*	Univariate	6	Poor
Naqvi et al. 1996 [21]	Pakistan	Retrospective Cohort	38	Mixed (13–75 years)	1990–1994	*P. falciparum*	Univariate	6	Poor

*P. falciparum: Plasmodium falciparum*; *P. vivax: Plasmodium vivax*.

### 3.2. Clinical manifestation

Our review included six clinical manifestations analyzed as prognostic factors in malaria patients with AKI: acute respiratory distress syndrome (ARDS), neurological involvement, disseminated intravascular coagulation (DIC), hypotension, oliguria, and ventilator requirement. The definitions or criteria for these variables were frequently inadequately specified in the included studies, and some varied across studies (Table S1). All of these factors showed significant associations with mortality risk (Table 2).

**Table 2.** Association between clinical manifestations and mortality risk in malaria patients with AKI.

Potential risk factors	Number of studies	OR	95% CI	*p* value	Heterogeneity (I^2^)
ARDS	5	18.50	6.80–50.37	<0.001	28.6%
Neurological Involvement	15	12.83	8.68–18.96	<0.001	0%
DIC	8	12.00	4.11–35.03	<0.001	66.3%
Hypotension	8	18.23	5.01–66.32	<0.001	55.4%
Oliguria	9	3.41	1.57–7.42	0.002	49.0%
Ventilator requirement	6	30.35	12.79–72.01	<0.001	0.2%

ARDS: acute respiratory distress syndrome; DIC: disseminated intravascular coagulation.

Among clinical manifestations, five studies analyzed the association between ARDS and mortality [6,7,12,15,19]. The pooled data showed that ARDS was significantly associated with mortality (OR: 18.50; 95% CI: 6.80–50.37; *p* < 0.001). Heterogeneity across studies was not statistically significant (I^2^ = 28.6%, *p* = 0.23), and the robustness of the results, confirmed by leave-one-out sensitivity analysis (Figure S1), underscores the importance of these findings.

In total, 15 studies analyzed the association between neurological involvement and mortality [6–15,19–21]. Neurological involvement included cerebral malaria, while some studies included only impaired consciousness or coma, assessed using or defined by a low Glasgow Coma Scale (GCS). Studies by Prasad R and Thanachartwet V included these variables in their analyses, resulting in a total of 15 datasets. The pooled result showed that neurological involvement was significantly associated with mortality (OR: 12.83; 95% CI: 8.68–18.96; *p* < 0.001). There was an absence of heterogeneity among the analyzed studies (I^2^ = 0% *p* = 0.46). Leave one out sensitivity analysis confirmed the robustness of the result (Figure S2). Beggs and egger’s test found no publication bias (Figure S3). After removing duplicate cohorts, the result remained significant (Figure S24).

Eight studies analyzed the association between DIC and mortality [7,8,11,12,14,15,19,20]. The pooled data revealed that DIC was associated with mortality (OR: 12.00; 95% CI: 4.11–35.03; *p* < 0.001). Substantial heterogeneity was found between studies (I^2^ = 66.3%; *p* = 0.0041). Leave one out sensitivity analysis showed that the result was robust and the study by Nigam, A.K became a main source of heterogeneity (Figure S4).

Eight studies analyzed the association between hypotension and mortality [6,7,11,12,15,16,18,19]. The pooled data revealed that hypotension was significantly associated with mortality (OR: 18.23; 95% CI: 5.01–66.32; *p* < 0.001). Notable heterogeneity existed between studies (I^2^ = 55.4%; *p* = 0.02). Leave one out sensitivity analysis confirmed the robustness of the result (Figure S5).

Nine studies analyzed the association between oliguria and mortality [7,9–12,15,17,19,20]. The pooled analysis revealed that oliguria was significantly associated with mortality (OR: 3.41; 95% CI: 1.57–7.42; *p* = 0.002). There was significant heterogeneity among the studies (I^2^ = 49.0%, *p* = 0.04). A leave-one-out sensitivity analysis showed that the result was robust, and omitting the study by Gupta BK reduced the heterogeneity to a non-significant level (Figure S6). After removing duplicate cohorts, the result remained significant (Figure S25).

Six studies analyzed the association between ventilator requirement and mortality [6,9,12,13,16,18]. The pooled analysis revealed that ventilator requirement was significantly associated with mortality (OR: 30.35; 95% CI: 12.79–72.01; *p* < 0.001). There was no significant heterogeneity, with a near absence of heterogeneity between studies (I^2^ = 0.2%, *p* = 0.41). Leave one out sensitivity analysis confirmed the robustness of the result (Figure S7).

The raw pooled forest plots for each analyzed factor are presented in the Supplementary Figures S8–S13. We also performed subgroup analysis based on study design, Plasmodium type, study quality, and participant age for the variables ARDS, DIC, hypotension, neurological involvement, oliguria, and ventilator requirement, presented in the Supplementary Figures S28–S51.

### 3.3. Laboratory manifestation

There were six laboratory manifestation factors analyzed for mortality risk in malaria patients with AKI, including leukocytosis, hyponatremia, anemia, acidosis, hyperkalemia, and jaundice. Similar to the previously analyzed variables, the thresholds or definition for these parameters were predominantly not specified in the included studies (Table S2). Except for anemia, all factors demonstrated conclusive results (Table 3).

**Table 3.** Association between laboratory manifestation and mortality risk in malaria patients with AKI.

Potential risk factors	Number of studies	OR	95% CI	*p* value	Heterogeneity (I^2^)
**Leukocytosis**	**3**	**4.87**	**1.70–13.89**	**0.003**	**12.5%**
**Hyponatremia**	**6**	**3.67**	**1.01–13.31**	**0.048**	**83%**
Anemia	3	6.92	0.75–63.66	0.088	63%
**Acidosis**	**3**	**4.88**	**2.42–9.84**	**<0.001**	**0%**
**Hyperkalemia**	**4**	**3.99**	**1.88–8.47**	**<0.001**	**2%**
**Jaundice**	**9**	**5.03**	**1.88–13.49**	**0.001**	**65%**

Three studies analyzed the association between leukocytosis and mortality among laboratory manifestations [12,13,19]. The pooled data showed that leukocytosis was associated with mortality (OR: 4.87; 95% CI: 1.70–13.89; *p* = 0.003). There was insignificant heterogeneity among the studies (I^2^ = 12.5%, *p* = 0.38).

Six studies analyzed the association between hyponatremia and mortality [7,9,12,14,19,20]. The pooled result showed that hyponatremia was significantly associated with mortality (OR: 3.67; 95% CI: 1.01–13.31; *p* = 0.048). There was significant heterogeneity between studies (I^2^ = 83%, *p* < 0.001). Leave one out sensitivity analysis showed that the result was not robust (Figure S14). After removing duplicate cohorts, the result remained significant (Figure S26).

Three studies analyzed the association between anemia and mortality [7,11,14]. The pooled analysis showed that anemia was not significantly associated with mortality (OR: 6.92; 95% CI: 0.75–63.66; *p* = 0.08). No significant heterogeneity was observed among studies (I^2^ = 63%, *p* = 0.07). The insignificance of our pooled final meta analysis result could be caused due to variations in the included population where 1 study was performed in adult, 1 in children, and the other one was performed in mixed populations, which weakened the pooled findings.

Three studies analyzed the association between acidosis and mortality [6,7,13]. The pooled data showed that acidosis was significantly associated with mortality (OR: 4.88; 95% CI: 2.42–9.84; *p* < 0.001). No evidence of heterogeneity was identified between studies (I^2^ = 0%, *p* = 0.42).

Four studies analyzed the association between hyperkalemia and mortality [7,9,12,13]. The pooled result showed that hyperkalemia was significantly associated with mortality (OR: 3.99; 95% CI: 1.88–8.47; *p* < 0.001). Heterogeneity among the studies was not significant (I^2^ = 2%, *p* = 0.38).

Nine studies analyzed the association between jaundice and mortality [7–9,11,12,14,19–21]. The pooled data showed that jaundice was significantly associated with mortality (OR: 5.03; 95% CI: 1.88–13.49; *p* = 0.001). There was substantial heterogeneity across studies (I^2^ = 65%, *p* < 0.001). Leave one out sensitivity analysis showed that the result was robust (Figure S15). After removing duplicate cohorts, the result remained significant (Figure S27).

The raw pooled forest plots corresponding to these analyses can be found in Supplementary Figures S16–S21. We also performed subgroup analysis based on study design, Plasmodium type, study quality, and participant age for the variables hyponatremia and jaundice, presented in the Supplementary Figures S52–S59.

### 3.4. Factors independently associated with mortality

We performed subgroup analyses based on the data reported (univariate vs multivariate odds ratios). Only 1 study reported their analysis using multivariate odds ratios [13]. Factors that were reported to be independently associated with mortality were ventilator requirement (OR: 10.806; 95% CI: 2.569–45.459) and leukocytosis (OR: 3.982; 95% CI: 1.146–13.836) (Supplementary Figures S22, S23). Other studies only analyzed their data using univariate measures (Table 1).

## 4. Discussion

The present meta-analysis of 16 studies was carried out to provide evidence about prognostic factors in malaria patients with AKI. Our findings identify several prognostic factors associated with mortality; however, the strength and consistency of these associations varied across variables. Factors such as ARDS, neurological involvement, ventilator requirement, acidosis, and hyperkalemia demonstrated more consistent and robust associations, whereas others should be interpreted with greater caution. Importantly, our meta-analyses do not provide evidence that anemia is the risk factor for mortality in malaria patients with AKI.

Notably, hyponatremia demonstrated substantial heterogeneity and lack of robustness in sensitivity analysis, suggesting that this association may be unstable. Therefore, it should be interpreted as a potential rather than definitive prognostic factor. Similarly, DIC, hypotension, and jaundice showed moderate to high heterogeneity, indicating variability in effect estimates across studies.

Multitude of factors and mechanisms underlie the development of AKI in malaria patients. Sequestration and cytoadherence of parasitized red blood cells in the endothelial cells are often theorized to be the leading causes [23]. Pathologies caused by aberrant immune responses are also considered as contributing factors [24]. Both the parasite and host response contributed to elicit damage in renal tissues, causing histopathological manifestations such as acute tubular necrosis [25]. As renal tissues are damaged, the physiological functions of the kidney will also be impaired, which causes a plethora of other abnormalities such as oliguria, elevated creatinine, uremia, acidosis, hyperkalemia, and hypernatremia.

Oliguria could indicate more extensive damage to kidney structures and functions, which might indicate severity and cause death, as reported in some patients with critical illnesses [26,27]. It can also indicate a delay in seeking medical attention, paving the way for other complications. Moreover, as the kidney function keeps declining, its ability to maintain electrolyte balance is also compromised [28], as well as its ability to reabsorb bicarbonate, resulting in acidosis [29]. Severe electrolyte imbalances are known to cause heart arrhythmia, a potentially fatal complication [30]. While acidosis can happen because of systemic hypoxia due to parasite sequestrations that block microvasculature, the co-existence of acidosis and AKI is a well-known medical emergency that requires urgent hemodialysis [31].

Neurological involvement, especially in patients with severe malaria, is a well-known and dreaded manifestation [5]. Although the cause of neurological involvement in malaria patients with AKI is difficult to identify, as parasite sequestration and uremia can have detrimental effects on the brain [32]. Therefore, the appearance of neurological manifestations might indicate direct damage to the brain or more severe damage to the kidneys, both of which are known to cause death.

Respiratory complication, including ARDS, might indicate fluid overload and/or aberrant immune activation, an increase in vascular permeability, or parasite sequestration in the pulmonary microvasculature. These process impair gas exchange, causing systemic hypoxia, which requires urgent care [33].

Our findings indicate that bilirubin is associated with mortality. This is caused by extensive schizogony, which causes damaged red blood cells to be phagocytized in the reticuloendothelial system. The heme in the red blood cells is transformed into bilirubin, which causes jaundice [34]. Due to its toxic effect on renal cells, bilirubin can also cause bile cast nephropathy [35]. Therefore, the presence of jaundice might indicate a higher parasite load or more extensive kidney damage.

Host factors are also important contributors toward the prognosis of malaria. Although never described in previous guidelines, we found that leukocytosis is associated with mortality. In general, malaria is typically associated with lower leukocyte counts compared with non-malarial conditions [36]. Leukocytosis is relatively uncommon in malaria but occurs more frequently in severe cases [37]. Consistent with previous studies, the presence of leukocytosis appears to reflect a poorer prognosis [38]. This finding may reflect dysregulation of the inflammatory response associated with severe malaria or the presence of secondary infection, both of which may complicate clinical management [39]. Bacterial co-infection, particularly bacteremia, is another plausible explanation of leukocytosis in malaria and is more common in severe cases, especially in children. The relationship between bacteremia and malaria is bidirectional. Severe co-infection may reduce parasite density and disease severity, whereas high parasitemia may increase bacteremia risk through intensified organ sequestration [40]. However, one study suggested that leukocytosis is an unreliable marker of bacteremia in malaria, being observed in approximately 4% of cases, all of which were severe, and only up to 14.3% in cases with bacterial co-infection [41]. Despite this potential association, we cannot exclude confounding by bacterial co-infection in our analysis. Methodological approaches varied across studies: Kaushik et al. [12] administered empirical antibiotics until resolution, Thanachartwet et al. [13] excluded mixed infections without specifying the method, and Kanodia et al. [19] excluded patients with other identifiable causes of fever and jaundice. Nevertheless, further studies are still needed to confirm the significance of this finding.

Hypotensive shock in malaria can occur due to dehydration or systemic vasodilation. It is a serious medical emergency that requires urgent resuscitation because these conditions impair tissue perfusion [42]. A delay in identifying hypotensive shock causes systemic damage, which has a grave prognosis by itself.

Disseminated intravascular coagulation is a well-known manifestation of aberrant inflammation in malaria. It causes systemic coagulation in all vasculature, worsening the tissue ischemia already caused by parasite sequestration [43]. Bleeding in other places also compromises hemodynamic functions, causing further systemic damage.

However, this current meta-analysis shows that anemia was not significantly associated with mortality. Considering the lack of studies that provide data on anemia, even though theoretically it is supposed to be significant. Anemia in malaria occurs due to the increased removal of circulating erythrocytes and decreased production of erythrocytes in the bone marrow; this leads to significant hemolysis, decreasing overall red blood cell counts [44]. This underscores the urgent need for further studies to confirm the significance of anemia in malaria patients with AKI.

There are few limitations of this meta-analysis that should be considered before interpreting the results. First, nearly half of the included studies were classified as poor quality, primarily due to inadequate adjustment for confounding factors. This raises the possibility of residual confounding and overestimation of effect sizes. Therefore, the findings should be interpreted with caution despite consistency observed in several analyses. Furthermore, only 1 study reported their data using adjusted measures, therefore limiting the applicability of our results. Second, variability in AKI definitions and the frequent lack of clear criteria for clinical and laboratory variables may have introduced unrecognized heterogeneity into the analysis (Table S2). Consequently, it was difficult to identify or explain why specific studies became sources of heterogeneity in leave-one-out sensitivity analysis with substantial heterogeneity (e.g., DIC, hypotension, hyponatremia, anemia, and jaundice). Although these sources could not be clearly determined based on variable definitions alone, further subgroup analyses were conducted for DIC, hypotension, hyponatremia, and jaundice to explore potential heterogeneity related to suspected confounding factors. The results varied, with heterogeneity predominantly attributable to mixed sources (patient characteristics or *Plasmodium* species) and the overall poor quality of included studies. However, duplicate cohorts did not significantly influence heterogeneity in the analyses of hyponatremia and jaundice. Nevertheless, our sensitivity analysis demonstrated that the results were generally robust despite the aforementioned definitional issues, including in neurological involvement. We also did not find newer literature after 2018, as we found that newer literature tended to include all types of severe malaria instead of exclusively including malaria with AKI [45,46]. Finally, we could not find any studies from the African region that met our selection criteria, limiting the generalizability of our results only to the included regions in our analysis. This underrepresentation may primarily result from our restriction to English-language publications and reliance on major databases. Although West and Central Africa bear the highest global *Plasmodium falciparum* burden, a substantial proportion of their medical literature is published in French, and many African journals are not indexed in PubMed or Scopus. Despite expanding our search to include WHO-IRIS and Embase, no eligible studies were identified. Malaria-associated AKI in sub-Saharan Africa differs substantially from predominantly Asian cohorts due to higher *Plasmodium falciparum* transmission, greater disease burden, and unique host factors such as malnutrition and genetic traits. These differences are further compounded by limited healthcare resources in high-burden African settings, including delayed presentation, nephrotoxin exposure, and restricted access to dialysis [47,48]. Consequently, findings from Asian cohorts may not accurately reflect malaria-associated AKI outcomes in these high-burden regions. Differences in epidemiology, parasite distribution, and healthcare systems may affect the applicability of our results to other regions.

## 5. Conclusion

In conclusion, several factors were associated with mortality in malaria patients with AKI, with ARDS, neurological involvement, ventilator requirement, acidosis, and hyperkalemia showing the most consistent and robust associations. Other factors, including hyponatremia, DIC, hypotension, and jaundice, should be interpreted with caution due to heterogeneity and variability across studies. The findings underscore the importance of heightened awareness and vigilance in managing patients with these conditions. Further studies that include all of these factors to better understand the interplay of them are advised to be performed. Given that the current analysis is largely based on univariate estimates and limited geographical data, these findings should be considered exploratory.

## Supplementary Material

Links Towards Supplementary Figures.docx

## Data Availability

Any supporting data related to our results will be made available upon request from the corresponding author.

## Collaborating authors or study group members

There are no collaborating authors for this review, and none of the authors are associated with any known study group.
