# Supplementary material for: Prognostic factors in malaria patients with acute kidney injury: a systematic review and meta analysis
Source: Ren Fail. 2026 May 25;48(1):2665050. doi: 10.1080/0886022X.2026.2665050 (PMC13202657; doi:10.1080/0886022X.2026.2665050)
Supplement: Links Towards Supplementary Figures.docx [file IRNF_A_2665050_SM6548.docx]

Dear

Editor-in-chief

Renal Failure

Dr Tibor Fülöp

We found that the size of the data of our supplementary figures causes our manuscript PDF to become unable to be processed. As suggested by the editorial office, we have uploaded our underlying data to the repository Figshare.

Should the editors and reviewer need to access the supplementary figures, please access via the following DOI link:

<https://doi.org/10.6084/m9.figshare.32012340>

We thank the editors and reviewers for their appreciation of our work. We hope the supplementary figures could be accessed properly.

Yours sincerely,

On behalf of all authors
